# Short report: self-reported psychopathic traits in Finnish and Dutch samples of non-referred adolescents: exploration of cultural differences

**DOI:** 10.1186/s13034-015-0090-3

**Published:** 2016-02-08

**Authors:** Svetlana Oshukova, Riittakerttu Kaltiala-Heino, Sanne Hillege, Corine de Ruiter, Grigori Joffe, Jouko Miettunen, Riikka Marttila, Mauri Marttunen, Matti Kaivosoja, Nina Lindberg

**Affiliations:** Psychiatry, Helsinki University and Helsinki University Hospital, P.O. Box 282, 00029 HUS, Helsinki Finland; School of Medicine, University of Tampere, 33014 Tampere, Finland; Department of Adolescent Psychiatry, Tampere University Hospital, 33380 Pitkäniemi, Finland; Child and Adolescent Psychiatry, VU University Medical Centre, Amsterdam, The Netherlands; Faculty of Psychology and Neuroscience, Maastricht University, Maastricht, The Netherlands; Research Unit of Clinical Neuroscience, Department of Psychiatry, Oulu University and Oulu University Hospital, Oulu, Finland; Medical Research Center Oulu, Oulu University Hospital and Oulu University, Oulu, Finland; Center for Life Course Health Research, Oulu University, Oulu, Finland; Adolescent Psychiatry, Helsinki University and Helsinki University Hospital, P.O. Box 590, 00029 HUS, Helsinki Finland; Mental Health Unit, National Institute for Health and Welfare, P.O. Box 30, 00271 Helsinki, Finland; Department of Child Psychiatry, Turku University, 20014 Turku, Finland; Hospital District of Central Ostrobothnia, Mariankatu, 16-20, 67200 Kokkola, Finland; Forensic Psychiatry, Helsinki University and Helsinki University Hospital, 04500 Kellokoski, Finland

**Keywords:** Adolescents, Cultural differences, Psychopathic traits

## Abstract

**Background:**

Culture-related differences in psychopathic traits have been reported for adults, but for adolescents such knowledge is lacking. The aim of this cross-national study was to compare self-reported psychopathic traits between Finnish and Dutch samples of mid-adolescent community youth.

**Methods:**

The Youth Psychopathic traits Inventory (YPI) was filled in by 372 Finnish and 474 Dutch 15- to 16-year-old pupils. As gender-specific differences exist in psychopathic traits, we analyzed the data separately for boys and girls.

**Results:**

Dutch boys scored significantly higher than Finnish boys on total and all dimensional scores of the YPI as well as on most sub-dimensional scores. Dutch girls scored significantly higher than Finnish girls on the Affective dimension and on the two corresponding sub-dimensions: remorselessness and callousness. Finnish girls scored significantly higher on grandiosity, which loads to the Interpersonal dimension of the YPI.

**Conclusions:**

Our findings suggest that culture influences the manifestation of psychopathic traits already in adolescence and that this relation is more prominent in boys.

## Background

Psychopathy is a constellation of interpersonal (dishonest charm, grandiosity, lying, and manipulative behavior), affective (remorselessness, unemotionality, and callousness), and behavioral (thrill-seeking, impulsivity, and irresponsibility) character traits [1]. Current conceptualizations see psychopathic traits on a dimensional continuum, where psychopathy is a malicious version of the extremes of normal personality traits [2]. Psychopathic traits are relatively stable over time, from childhood through adolescence to adulthood [3]. The gold standard for assessing adolescent psychopathic traits is the Psychopathy Checklist-Youth Version (PCL-YV) [4], which is an adaptation of the Psychopathy Checklist-Revised [5], designed for adults. The PCL-YV is, however, a time-consuming method that demands rigorous training and is mainly used in forensic samples. Because of this, various self-assessments have been widely used to measure psychopathic traits. Self-assessments are also cost effective to screen large samples. However, lack of valid cut-off points and valid reference groups limit their use in clinical practice.

In light of current literature, psychopathy is strongly associated with genetic and neurobiological background [6–8], but environmental factors also have an influence on its development [9]. Culture refers to the set of socially constructed and learned norms, values, beliefs, and behaviors shared by a group of individuals. Among adults, a recent community sample study covering 58 nations and more than 33,000 adults found that males and females from Western Europe produced the highest scores on the affective facet of the Self-Report Psychopathy scale, while individuals from Northern Europe tended to exhibit the lowest scores on both the affective and interpersonal facets [10]. With regard to juvenile psychopathy, as far, the main priority in psychopathy research has been to establish the validity of existing conceptualizations of juvenile psychopathy rather than to examine the construct in relation to culture. However, culture can influence juvenile antisocial behavior by operating through larger social systems [11]. Although psychopathy and antisocial behavior are distinct constructs, individuals with elevated psychopathic traits often exhibit disruptive behavior [12], and the behavioral component of the Psychopathy Checklist-Revised [5] highly reflects antisocial lifestyle.

The aim of this cross-national study was to compare psychopathic traits between Finnish and Dutch samples of 15- to 16-year-old non-referred youth. As gender-specific differences exist in psychopathic traits [13], we analyzed the data separately for boys and girls. We hypothesized that, in line with the recent study by Neumann et al. [10] among adults, Dutch adolescents representing Western Europe, would exhibit higher levels of psychopathy than Finnish adolescents representing Northern Europe.

## Methods

### Subjects

#### The Finnish sample

The sample comprised adolescents attending the 9th grade at five Finnish-speaking secondary schools in Kokkola city with approximately 47,000 citizens, on the western coast of Finland. Of the 446 students, 60 (13.4 %) did not participate in the study because of either failing to attend school on the study day or refusing to participate. Of the remaining 386 students, five did not complete the self-assessment and six did not answer the questions on general background variables, and thus, were excluded. From the 375 adolescents, those who were aged 15–16 years at the time of the assessment were selected. Thus, the final sample comprised 372 adolescents, 174 boys (46.8 %) and 198 girls (53.2 %), with a mean age of 15.1 years (SD = 0.27). For details, see [13].

#### The Dutch sample

The original sample comprised 776 adolescents in the upper grades of two secondary schools in two rural areas of the Netherlands. However, 36 adolescents (4.6 %) did not complete the questionnaire. From 740 pupils, those of foreign origin (e.g., Somalian, Turkish, Netherlands-Antillean) (n = 74, 10.0 %) as well as those younger than 15 years or older than 16 years (n = 238, 32.2 %) were removed from the sample. Thus, the final sample included 474 adolescents, 221 boys (46.6 %) and 253 girls (53.4 %), with a mean age of 15.4 years (SD = 0.49). For details, see [14].

### Procedure

The adolescents filled in the self-assessment during their school lessons. Prior to completing the assessments, they received both oral and written information about the study. In Finland, return of the completed questionnaires by the participants was taken as confirmation of their consent. Privacy was ensured by having no identifying factors in the questionnaires, only age and gender were collected as background variables. In the Netherlands, written informed consent was obtained from all participants. In both countries, a letter was sent to the guardians of the students to inform them about the study. The study was approved by the local ethics boards and the administration of the schools.

### The Youth Psychopathic traits Inventory (YPI)

The Youth Psychopathic traits Inventory (YPI) by Andershed et al. [15] was used to measure psychopathic traits. It assesses each psychopathic trait with several items, which are composed to tap psychopathic traits indirectly, framing the psychopathic features as abilities rather than deficits (e.g. “I usually feel calm when other people are scared” instead of “My emotions are shallow”). It consists altogether 50 statements scored on a 4-point Likert scale with response options ranging from “Does not apply at all = 1” to “Applies very well = 4”; thus, the total score of the scale can range from 50 to 200, with a higher score representing a higher level of the trait. The YPI has three dimensions and 10 sub-dimensions. The Interpersonal (Grandiose-manipulative) dimension contains sub-dimensions termed Dishonest charm, Grandiosity, Lying, and Manipulation, the Affective (Callous-unemotional) dimension contains Remorselessness, Unemotionality, and Callousness, and the Behavioral (Impulsive-irresponsible) dimension contains Thrill-seeking, Impulsiveness, and Irresponsibility. The original YPI showed internal consistencies ranging from marginal (Callousness: Cronbach’s alpha = 0.66; Unemotionality: 0.67) to acceptable and good (0.71–0.82) [15]. Later, and with various language versions, the YPI has shown moderate to good psychometric properties both in general [13, 14, 16, 17] and in forensic samples [18, 19]. In this study, we used the authorized Finnish [13] and Dutch [14] versions of the YPI. Both researcher groups used the English version of the YPI as a basis and performed the translation according to the recommendations of the developers (http://www.oru.se/jps/downloadYPI/) including an iterative process of translation and independent back translation, followed by a discussion to resolve minor differences.

### Statistical analyses

In order to evaluate the internal consistency of the YPI, we calculated Cronbach’s alphas for the total, dimensional and sub-dimensional scores in both samples. In line with previous research, reliability coefficients of <0.60 were interpreted as insufficient, 0.60–0.69 as marginal, 0.70–0.79 as acceptable, 0.80–0.89 as good, and ≥0.90 as excellent [20].

The Mann–Whitney U test was used to compare the YPI scores because the data was skewed. Both average and median continuous scores are reported in line with previous research [13–15, 21]. We used Cohen’s d to estimate the effect sizes of the differences, interpreting an effect size of 0.2–0.5 as small, 0.5–0.8 as medium, and >0.8 as large [20].

All statistical analyses were performed with IBM SPSS Statistics version 22.

## Results

### Internal consistency

In both genders, for the total score as well as for both the Interpersonal and Behavioral dimension score of the YPI, Cronbach’s alpha coefficients indicated good to excellent internal consistencies in the Finnish sample, but acceptable to good in the Dutch one. For the Affective dimension score, internal consistency was insufficient in Finnish boys, marginal in Dutch adolescents, and acceptable in Finnish girls. The internal consistencies were at least acceptable for most of the sub-dimensions, but there were some exceptions. The sub-dimension Callousness showed insufficient internal consistency in both samples across gender, and in the Dutch sample, Cronbach’s alpha coefficients revealed insufficient internal consistencies for the sub-dimension Unemotionality in both genders and for the sub-dimension Thrill-seeking in boys (Table 1).

**Table 1:** Internal consistencies (Cronbach’s alphas, α), descriptives, and group differences for the sub-dimensional, dimensional and the total scores of the Youth Psychopathic traits Inventory (YPI) in 15- to 16-year-old Finnish and Dutch boys and girls

	Internal consistency		Descriptives and group differences						
	Finnish boys	Dutch boys	Finnish boys (n = 174)		Dutch boys (n = 221)		Statistics		
	α	α	Mean (SD)	Median	Mean (SD)	Median	U	p	Cohen’s d
YPI sub-dimension									
Dishonest charm	0.81	0.74	1.81 (0.67)	1.80	2.02 (0.65)	2.00	15,325.50	0.001	−0.318
Grandiosity	0.79	0.81	1.89 (0.68)	1.80	1.86 (0.77)	1.75	18,074.50	NS	0.041
Lying	0.82	0.72	1.80 (0.67)	1.80	2.01 (0.65)	2.00	15,343.00	0.001	−0.318
Manipulation	0.86	0.81	1.70 (0.69)	1.60	1.98 (0.71)	1.80	14,399.00	<0.001	−0.440
Remorselessness	0.83	0.63	1.69 (0.64)	1.60	2.01 (0.61)	2.00	12,963.00	<0.001	−0.440
Unemotionality	0.65	0.50	2.15 (0.63)	2.20	2.24 (0.55)	2.20	1741.00	NS	−0.152
Callousness	0.41	0.25	2.23 (0.51)	2.20	2.52 (0.46)	2.40	13,308.50	<0.001	−0.440
Thrill-seeking	0.80	0.55	2.55 (0.72)	2.60	2.78 (0.57)	2.80	15,541.50	0.001	−0.353
Impulsiveness	0.73	0.62	2.14 (0.67)	2.00	2.37 (0.62)	2.40	15,298.00	<0.001	−0.440
Irresponsibility	0.74	0.60	1.85 (0.70)	1.80	1.85 (0.67)	1.80	18,782.00	NS	0.000
YPI dimension									
Interpersonal	0.90	0.85	7.19 (2.37)	7.00	7.88 (2.31)	7.40	15,686.50	0.002	−0.294
Affective	0.55	0.61	6.07 (1.30)	6.00	6.77 (1.24)	6.80	12,756.50	<0.001	−0.440
Behavioral	0.86	0.71	6.54 (1.84)	6.35	7.01 (1.44)	6.80	15,886.50	0.003	−0.285
YPI Total	0.84	0.74	19.81 (4.91)	19.60	21.66 (4.18)	21.25	14,360.00	<0.001	−0.440
	Finnish girls	Dutch girls	Finnish girls (n = 198)		Dutch girls (n = 253)		Statistics		
	α	α	Mean (SD)	Median	Mean (SD)	Median	U	p	Cohen’s d
YPI sub-dimension									
Dishonest charm	0.82	0.75	1.72 (0.66)	1.60	1.72 (0.60)	1.60	24,243.00	NS	0.000
Grandiosity	0.82	0.76	1.58 (0.61)	1.40	1.34 (0.48)	1.20	18,940.00	<0.001	0.437
Lying	0.77	0.78	1.64 (0.60)	1.40	1.64 (0.62)	1.40	24,980.50	NS	0.000
Manipulation	0.81	0.77	1.62 (0.63)	1.40	1.57 (0.57)	1.40	24,491.50	NS	0.083
Remorselessness	0.78	0.63	1.43 (0.52)	1.33	1.59 (0.52)	1.40	19,669.50	<0.001	−0.308
Unemotionality	0.65	0.57	1.79 (0.54)	1.70	1.71 (0.47)	1.60	23,036.00	NS	0.158
Callousness	0.44	0.46	1.67 (0.48)	1.60	1.89 (0.52)	1.80	18,583.00	<0.001	−0.440
Thrill-seeking	0.78	0.72	2.47 (0.65)	2.55	2.51 (0.64)	2.40	24,538.00	NS	−0.062
Impulsiveness	0.77	0.71	2.20 (0.67)	2.20	2.32 (0.64)	2.20	22,805.50	NS	0.183
Irresponsibility	0.68	0.59	1.60 (0.57)	1.40	1.56 (0.51)	1.40	24,746.00	NS	0.074
YPI dimension									
Interpersonal	0.87	0.83	6.56 (2.12)	6.20	6.28 (1.86)	5.80	23,538.00	NS	0.140
Affective	0.73	0.61	4.89 (1.24)	4.60	5.18 (1.11)	5.00	20,389.00	0.001	−0.246
Behavioral	0.80	0.76	6.27 (1.59)	6.20	6.39 (1.47)	6.20	23,852.00	NS	−0.078
YPI Total	0.77	0.76	17.71 (4.18)	17.10	17.84 (3.72)	17.20	24,342.00	NS	−0.032

Comparisons are performed using the Mann–Whitney U test. Effect sizes are reported

*NS* not statistically significant

### Psychopathy scores

**Fig. 1:**
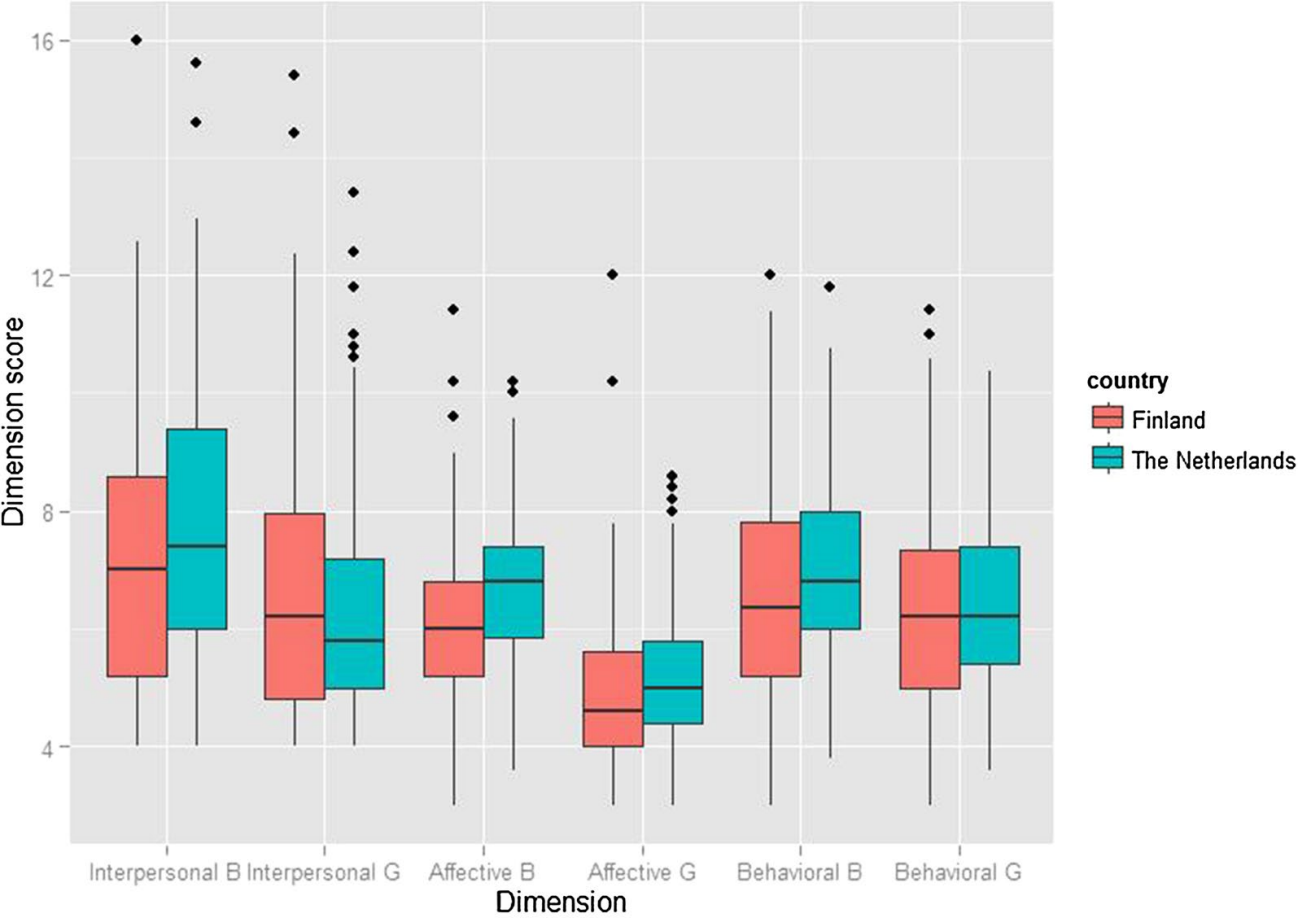
The Youth Psychopathic traits Inventory (YPI) dimension scores in 372 Finnish and 474 Dutch adolescents aged 15–16 years. *B* boys, *G* girls

The Dutch boys scored significantly higher than the Finnish boys on the YPI total score and all dimensional scores as well as on sub-dimensional scores except Grandiosity, Unemotionality, and Irresponsibility, where no significant differences were observed (Table 1; Fig. 1). The Dutch girls scored significantly higher than their Finnish counterparts on the Affective dimension score and on the two corresponding sub-dimensions: Remorselessness and Callousness. The Finnish girls, on the other hand, scored significantly higher on Grandiosity.

## Discussion

Most previous studies on the association of ethnicity and culture with adolescent psychopathic traits have focused on differences across race [22, 23] or between Europeans and ethnic minorities, mostly among offender samples [24, 25]. There has been some evidence that African-American youth tend to score higher on psychopathic traits than European-American youth [22], and that psychopathic traits influence criminal behavior in different ways across race [23]. On the other hand, neither Pechorro et al. [24] nor Veen et al. [25] found differences in manifestations of psychopathic traits between ethnic groups. To our knowledge, this study is the first to compare community youth of two European countries on level of psychopathic traits.

Dutch boys exhibited significantly higher psychopathic traits than their Finnish peers. With regard to dimensions, they also showed higher levels of affective and interpersonal traits of the psychopathy construct. Dutch girls, on the other hand, scored significantly higher than Finnish girls only on the Affective dimension. This finding is interesting, since, according to many researchers, it is interpersonal and affective features that comprise the “core” of the psychopathic character, and the behavioral component could only be seen as a consequence of the syndrome [26]. Our findings definitely concur with those from a recent world-wide community research by Neumann et al. [10] among adults , and suggest, as we hypothesized, that culture- related differences in psychopathic traits can be detected already in adolescents.

Dutch boys also scored significantly higher than Finnish boys on the behavioral component of psychopathic traits, reflecting impulsive and irresponsible lifestyle, while among girls this difference was not observed. Interestingly, Pickett et al. [27] explored self-reported physical fighting among school boys and girls in 35 different countries. Involvement of boys in physical fighting during the previous year was lowest in Finland (36.7 %), while the Netherlands ranked 9th (50.5 %). However, Dutch girls also reported being involved in fighting more often than their Finnish peers (22.1 vs. 13.3 %), which is not in line with our results.

An obvious strength of this study was that we were able to collect two samples of mid-adolescent community youth evaluated with an instrument (YPI) widely used in psychopathy research with adolescent populations. However, the samples were not collected simultaneously, but as two individual study projects: the Dutch-sample was collected in 2005 and the Finnish one in 2014. Since the Finns, contrary to the Dutch, are an ethnically extremely homogeneous population, we excluded adolescents of foreign origin. Although the YPI has often been used in community youth, concern has risen on its ability to measure the affective component of the psychopathy construct [21]. In line with many previous community studies [15–17], we found especially low internal consistency coefficients for the Unemotionality and Callousness sub-scales. It has been proposed that these sub-scales contain too few items to permit adequate scale reliabilities and that the YPI should be revised in the future [21]. So, weak scale reliabilities of some sub-scales observed in our study rather reflect the known weakness of the questionnaire than indicate that the translated versions might not be culture-adequate. Also, the effect sizes were small pointing to a sufficient content validity of the translated YPI versions and, to some extent, a true culture-effect.

## Conclusions

Our cross-national study suggests culture-related differences in juvenile psychopathic traits. This preliminary research should obviously be replicated with other cross-national samples; if significant differences emerge, the YPI as well as other self-report questionnaires for psychopathic traits might need nation-specific reference values. At present, some caution is needed in generalizing the national research findings.
